# Pseudo contrastive labeling for predicting IVF embryo developmental potential

**DOI:** 10.1038/s41598-022-06336-y

**Published:** 2022-02-15

**Authors:** I. Erlich, A. Ben-Meir, I. Har-Vardi, J. Grifo, F. Wang, C. Mccaffrey, D. McCulloh, Y. Or, L. Wolf

**Affiliations:** 1 The Alexender Grass Center for Bioengineering, School of Computer Science and Engineering, Hebrew University of Jerusalem, Jerusalem, Israel; 2 Fairtilty Ltd., Tel Aviv, Israel; 3 Infertility and IVF Unit, Department of Obstetrics and Gynecology, Hadassah Ein-Kerem Medical Center and Faculty of Medicine, Hebrew University of Jerusalem, Jerusalem, Israel; 4 Fertility and IVF Unit, Department of Obstetrics and Gynecology, Soroka University Medical Center and the Faculty of Health Sciences Ben-Gurion University of the Negev, Beer-Sheva, Israel; 5 New York University Langone Prelude Fertility Center, New York, NY USA; 6 Fertility and IVF Unit, Obstetrics and Gynecology Division, Kaplan Medical Center, Rehovot, Israel; 7 The School of Computer Science, Tel Aviv University, Tel Aviv, Israel

**Keywords:** Computational biology and bioinformatics, Biomedical engineering

## Abstract

In vitro fertilization is typically associated with high failure rates per transfer, leading to an acute need for the identification of embryos with high developmental potential. Current methods are tailored to specific times after fertilization, often require expert inspection, and have low predictive power. Automatic methods are challenged by ambiguous labels, clinical heterogeneity, and the inability to utilize multiple developmental points. In this work, we propose a novel method that trains a classifier conditioned on the time since fertilization. This classifier is then integrated over time and its output is used to assign soft labels to pairs of samples. The classifier obtained by training on these soft labels presents a significant improvement in accuracy, even as early as 30 h post-fertilization. By integrating the classification scores, the predictive power is further improved. Our results are superior to previously reported methods, including the commercial KIDScore-D3 system, and a group of eight senior professionals, in classifying multiple groups of favorable embryos into groups defined as less favorable based on implantation outcomes, expert decisions based on developmental trajectories, and/or genetic tests.

## Introduction

In vitro fertilization (IVF) is the most effective form of assisted reproductive technology. However, IVF treatments are inefficient, costly, lengthy, and place an emotional burden on the future parents. In particular, selecting the embryos with the best implantation potential remains a challenging task. Despite decades of clinical practice, there is no reliable non-invasive method to identify the small fraction of embryos that possess the highest potential to develop into a blastocyst, which can then be implanted and hopefully proceed to term^1^. Thus, to maintain reasonable pregnancy rates, current practice often involves the transfer of multiple candidate embryos into the uterus. This results in clinical complications and health risks to both the newborn and the mother, with over 10% of IVF pregnancies resulting in twins or more^2^.

Several methods have been proposed to rate embryos by their implantation likelihood. Typically, a single image of the embryo is taken by a microscope mounted on a camera moments before transferring, typically at day 5^3^. Methods for earlier development stages are rarely studied, perhaps because it is considered almost impossible to identify viable embryos based on a single pre-blastocyst image^4,5^.

Time-lapse incubation (TLI) has the potential to improve identification based on the availability of additional data points and the possibility of modelling embryo development. Time lapse videos typically consist of 70 images/day, with 7 focal planes each. That is, TLI provides approximately 2500 images for each day 5 blastocyst embryo. To date, there is no accepted or standardized algorithm that exploits the added information in the TLI sequence. Notably, none can outperform traditional, manual microscopy-based scoring. As a result, despite the added information provided by TLI, IVF pregnancy rates remain low.

TLI studies are thus often focused on later developmental stages, mainly day 5 blastocysts, and require manual annotations of numerous spatial-temporal features from the TLI stream to obtain a single score^6^. To the best of our knowledge, our method is the only one that can be applied to all embryos at any time or developmental stage.

Our work integrates classification information over time to benefit from the availability of multiple TLI-originated images during training, and can be applied to either TLI image streams or single microscopy images during test. We do not aim to track or meet explicit developmental milestones. Instead, we focus on obtaining a stable classification score by building shared feature layers for the multiple development stages and making use of unlabeled or ambiguously labeled embryos. The latter is crucial, since many embryos are not transferred, and even those that are, tend to be typically transferred as part of a group of embryos, for which the outcome of each individual embryo is uncertain.

Our semi-supervised method is based on assigning pseudo-ranking labels to pairs of embryos. A classifier is then trained based on these labels, by employing a Siamese network. Information is further integrated by considering multiple time points. The improved labels are then used to obtain a second set of pseudo labels and the process repeats one additional time.

Our results indicate that temporal integration helps, even if it is employed only during training, that employing pseudo labels together with pair contrasting is effective, and that the obtained method significantly outperforms current commercial systems.

## Related work

In the early days of IVF treatments, a two dimensional snapshot was taken by a microscope at the different development stages of the embryo^4^. Many machine based models have been proposed involving clinical features, such as oocyte age, causes of infertility, oocyte stimulation, and semen analysis, in addition to embryonic data^7,8^.

In particular, several (typically 10–100) features are chosen, which are then subjected to a feature selection technique such as SVM^9^, multivariate logistic regression^10^, genetic algorithm and/or decision trees^11^, Bayesian networks^12^ and even ANN^13^. Nevertheless, all these methods mainly concentrate on predicting the probability of a patient to conceive, rather than ranking which embryo has the best probability of implanting given a group of possible embryos and their corresponding images. Several models have been proposed for ranking embryos based on a single image^7,8^, taken at a specific time such as day 3, cleavage-stage^14^, day 5, the blastocyst stage^9^ or even day 1, when the pronuclei appear^15^.

To overcome the inability to accurately evaluate early stage embryos, culturing embryos to the blastocyst stage combined with grading protocols have frequently been implemented in both research and clinical practice^4,16–18^. Several AI methods were recently presented. STORK^19^ automates and ERICA^20^ replaces the manual ranking of late blastocyst embryos. To develop STORK , expert embryologists were asked to grade embryos using the Veeck and Zaninovich system^21^, a slightly modified version of the Gardner system^4^, as poor, fair or high quality. Then, a CNN was trained to distinguish between the high quality and the poor quality embryos, disregarding the fair quality embryos. They reported an AUC of 0.98 predicting whether an embryo was of good quality or poor quality over a test set tagged by one of the Cornell clinic’s embryologists. However when the embryos were tested by embryologists in a different hospital (Universidad de Valencia, Valencia) the AUC dropped to 0.75, suggesting that STORK overfitted to the blastocyst grading at the Cornell clinic. Moreover, fair quality^22^ accounts for more than 40% of the STORK data and is the hardest to identify. Finally, the STORK system implements a scoring system rather than attempting to predict implantation outcomes. Recent studies suggest that the Gardner score is not well aligned with implantation probability or even euploidy^20^. ERICA^20^ is a two-step method with the goal of ranking late stage blastocysts. In the first step, 96 spatial features are extracted from a 2D image. This is followed by a second step, in which a CNN is trained over these 96 features to predict embryo ploidy and implantation. Note that the spatial features are not precisely defined and require human expertise. ERICA was reported to obtain an accuracy of 70%. however, the train and test embryos were hand-picked to include “good” images; i.e. embryos of lower quality were ignored. Furthermore, the sample only consisted of 84 embryos from 19 patients and the test set was biased by a criterion of having at least one euploidy and one aneuploidy embryo. This may imply a skew towards younger patients^11,12,23^ since patient age was shown to predict implantation^23^. In addition, some of the embryos were tagged as positive based solely on beta human chorionic gonadotropin (beta-hCG) results, which is not sufficient, since implantation is normally defined as the presence of (at least) a gestational sac or a heartbeat^8,19^, to exclude early miscarriages (chemical pregnancy). More importantly, more than 95% of the embryos in their study were either in the middle of, or after the hatching stage, which typically corresponds to day 6 or day 7 embryos, while the vast majority of transfers in the current standard of care take place earlier.

Time-lapse incubation (TLI) enables the continuous tracking and screening of fertilized embryos, unlike traditional microscopy that is limited to snapshots of several discrete points in time^24,25^. Time-lapse imaging overcomes some of the drawbacks of traditional microscopy, such as exposing the embryos to environmental changes^26^. It is known that time-lapse microscopy can point to new dynamic markers of embryo quality and facilitate the creation of novel algorithms for effective embryo selection^27^.

Several algorithms have been proposed in the last few years based on embryo kinetics and morphology as identified manually by experts, and have been used to predict implantation^25,28,29^, blastocyst formation^29^ and even genetic chromosomal disorders^30,31^. However, time lapse incubators have yet to yield better prediction results than regular microscopy^27^. Moreover, current studies are based on morphology and morphokinetic parameters which require manual annotation and are thus associated with intrinsic variability.

A few studies have addressed the task of blastocyst formation but have mainly considered embryos that were observed for 5 days, by which time 97% of the blastocyst embryos had already developed into blastocysts^13^. These authors reported an AUC of 80% with the blastocyst scoring algorithm, which was found however, to be inferior to manual accuracy by visual inspection. Several studies have reported positive correlations between pronuclei markers and embryo viability. Specifically, markers such as the appearance of pronuclei and fading timing (tPNa, tPNf), the number of pronuclei, pronuclei shape, symmetry, and joint path were found to be associated with blastocyst development and implantation outcomes^32–34^. However, these markers have never been integrated into a scoring system, and not all of the embryos in these studies were placed in the TLI early enough to track the pronuclei stage.

Other studies have addressed the task of cleavage stage (day 3) classification using time lapse imaging^25,28^. However, they did not consider events after 76 h. A study by Liu et al.^35^ presented a flow diagram with six conditions for scoring the embryo with a grade ranging from A to F after 68 h.

The most common (patented) implantation scoring for cleavage stage embryos is EmbryoScope’s built-in algorithm (Vitrolife) called KIDScore-D3, which gives a (one-time) prediction after 66 h. KIDScore-D3 implements a binary decision tree that is based on five morphokinetic conditions. The authors report an AUC of 0.65 in predicting implantation over day 3 transfers^36^. Another built-in score, called KIDScore-D5 combines both morphokinetic with morphological assessment and provides a score between 1 and 6. A recent study^37^ suggested that the KIDScore-D5 improves implantation rates, compared to traditional morphological scoring, and is associated with PGT euploidy. It was not released to the public, so its performance cannot be evaluated. Crucially, all these studies are tailored for specific timing or are based on manually selected features that are only present in a small fraction of the TLI stream, thus making them hard to use in practice, given the high workload as well as high user expertise.

Other recent studies have suggested new markers and grading models for day 5 blastocyst embryos based on TLI tracking. One study reported a strong correlation between spontaneous blastocyst collapse and pregnancy outcomes^38^. In another study, it was shown that TLI streaming can be exploited to automatically classify Inner Cell Mass (ICM) and Trophectoderm grading^22^. The results indicated that TLI led to superior results compared to a single microscopy image snapshot. Another study employed different spatial parameters manually acquired at different time/development stages (mainly late blastocyst stages) to accurately predict implantation results^6^. However, there is still no quantitative scoring algorithm based on these findings.

The first deep network for predicting the implantation potential of blastocyst embryos from time-lapse videos was recently presented^39^. Unfortunately, the way the entire video sequence was fed to the network was not described in this study. The authors reported an AUC of 93%. However, considering the distribution of the data-set (694/1079/7063 KID-positive/KID-negative/Discarded), the results correspond to a model that intentionally excluded 100% of the discarded embryos and predicted almost randomly (with a 55% success rate) over all the remaining embryos (i.e., transferred embryos with KIDp/KIDn labels). Since by definition all the discarded embryos could have been discarded manually, these results reflect a 55% accuracy for the KIDp/KIDn embryos. Further, it could only be evaluated over blastocyst embryos, so this algorithm cannot be used as a grading system as of the early stages of embryonic development (days 0–3).

A more recent study^40^ employed an ensemble of spatial and temporal models to predict blastocyst formation and blastocyst quality based on a deep network framework. A massive data-set of 26,000 embryos from 2600 cycles was studied. However, after filtering embryos for different reasons, including not having been cultured to day 5, the authors were left with about 11,500 embryos. In addition, the target labels for embryo quality were not implantation results, but rather a majority vote by four experienced embryologists who determined whether a blastocyst was usable or not. Considering the difference between the cleavage time of IVF-fertilized embryos and ICSI-fertilized embryos, the authors suggested first synchronizing the embryo timing according to the time of pronuclei fading (tPNf). A single frame model was then trained to predict the number of cells per image, up to five. Finally, the output of this model was fed into an LSTM network, termed STEM, which predicted blastocyst formation, and STEM+, which predicted blastocyst quality. The spatial morphological model was trained using 1000 features per frame, extracted with DenseNet over 35 frames between day 1 and day 3 post-insemination, resulting in an output feature map of $35\times 1000$. The authors then applied a gradient boosting classifier to this map in order to predict blastocyst formation (STEM) and blastocyst quality (STEM+). To optimize the accuracy of the validation set, both models were jointly weighted. This approach used multiple images from the TLI; however, it only provides a single score rather than continuous scores over time.

Thus overall, most cleavage stage and blastocyst stage studies ignore the extra information that exists outside this particular developmental stage, and fail to utilize the majority of the images that the TLI collects. To date, there is no algorithm that exploits TLI streaming as a whole and translates continuous embryo streaming into continuous embryo scoring that is applicable to all embryos regardless of stage. By contrast, we present a simple approach that addresses these limitations. We propose a novel classifier training method that takes into account the time since fertilization for each frame. This classifier is then integrated over all existing frames to produce a score for each time point, providing a continuous evaluation of the embryo based on the TLI data.

## Method

For a single embryo, time-lapse imaging acquires seven layers of z-stack images $15\,\upmu \hbox {m}$ apart at each time point every 15 to 20 min, totaling 360–480 images over a 5-day period, for each focal plane, with time 0 the time of fertilization. Each of the seven focal planes represents a different focus of the embryo. A sample with all seven focals was used in our study as seven different training samples, resulting in an increase of seven fold in the size of our training dataset. This was done in order to increase the variability of the training data and to make the algorithm generalize better, i.e., as a form of augmentation. During test time, we opted for the central focal plane ($0\,\upmu \hbox {m}$), which is typically the most focused.

**Figure 1:**
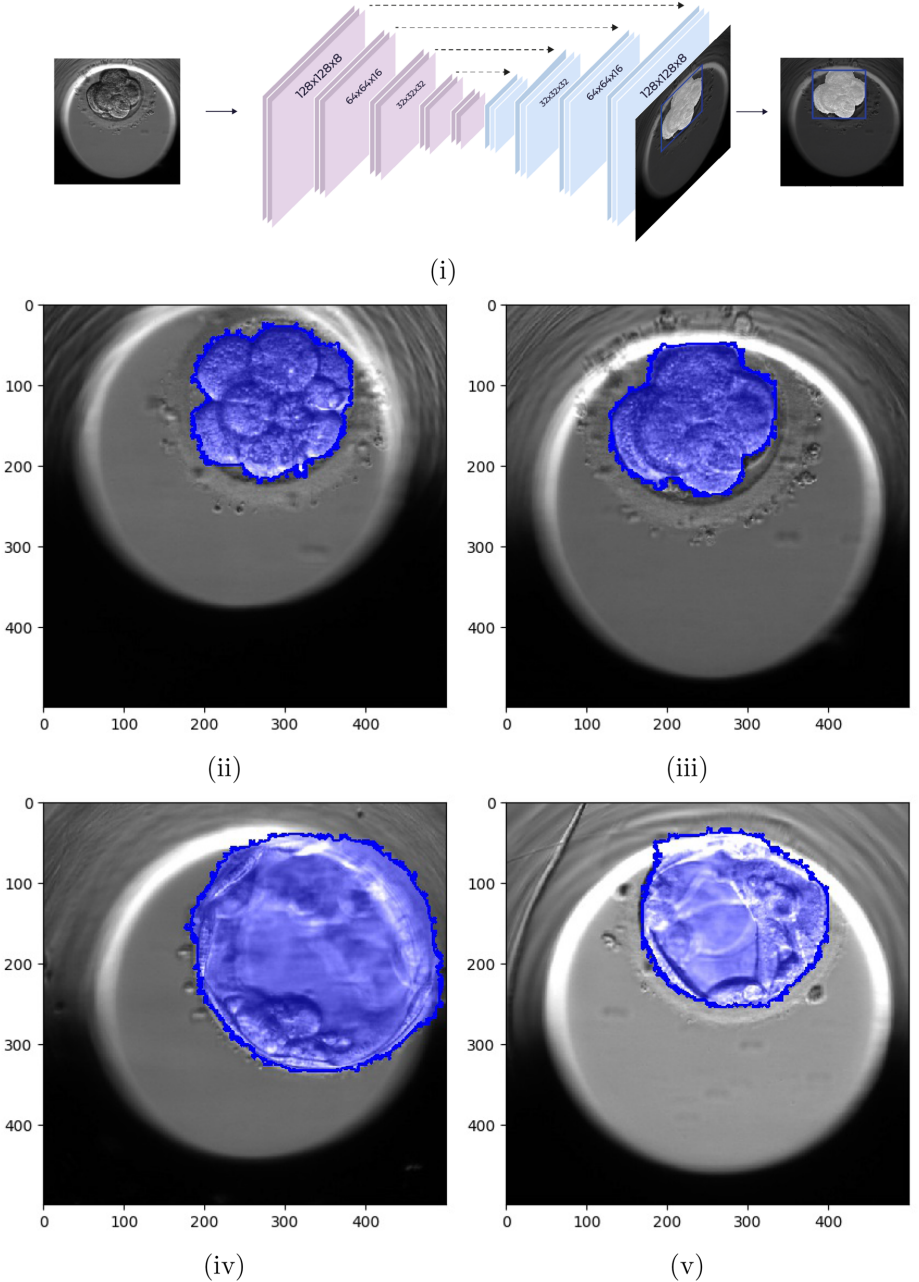
Embryo pixelwise segmentation using a UNet^41^. (**i**) U-NET architecture for embryo localization and segmentation. Input images are $500\times 500$, and outputs are segmentations of the embryo at pixel level. (**ii**–**iv**) Examples of network output masks based on developmental stage. (**ii**) 8 cells, (**iii**) 10 cells, (**iv**) blastocyst, (**v**) expanded blastocyst.

Each image ($500\times 500$ pixels) depicts a centered spheroid culture well of a size that is approximately $430\times 430$ pixels. The embryo itself can be found anywhere inside the well. Segmentation of the embryo sub-image is used for pre-processing by employing a UNet^41^ network trained to minimize a pixel-wise soft hinge loss function, see Fig. 1.

Since the embryo is subject to high variability due to temporal changes, from one cell to fully hatching, it is mandatory to accurately segment which pixels belong to the embryo. The imbalance between the “foreground” pixels (i.e, part of the embryo) and “background” pixels, and the varying difficulty between pixels of each type, call for a careful weighting of the training objective. Inspired by the focal loss^42^, our objective function multiplies two terms per pixel. The first is a binary soft hinge loss, which was shown to both smooth and upper bound the 0–1 loss^43^. This is obtained by performing the soft-max operation: $max_x f(x) \le (1/\gamma )\log \sum _x e^{xf(x)}$ over the hinge-loss used in Support-Vector-Machines (SVM)^44^ for sets of target *y* and prediction *s*, $l(s,y) = max(0,m-y\cdot s)$, where *m* is the margin parameter.

1 $$\begin{aligned} l_p(s_p,y_p) = \log \left( 1+e^{\gamma \left( m-y_p\cdot s_p\right) } \right) \end{aligned}$$

where $y_p$ is the mask ground-truth at pixel *p*, which denotes whether the pixel is part of the embryo , and $s_p$ is the prediction score at the pixel *p*. The margin *m* and the constant $\gamma$ are both set to 1.

The second term weights all pixels, such that pixels with scores that already correspond to their respective ground-truths are underweighted. To compensate for the imbalance between foreground and background, each type is separately weighted to have a 1 unit weight.

2 $$\begin{aligned} w_p(s_p,y_p)= \frac{e^{-s_p\cdot y_p}}{\sum _{q\in \left[ P \right] , y_q=y_p}e^{-s_p\cdot y_p}} \end{aligned}$$

The overall loss per single example is given by,

3 $$\begin{aligned} l= \sum _{p \in \left[ P \right] }w_p\left( s_p, y_p\right) \cdot l_p\left( s_p,y_p\right) \end{aligned}$$

The training was performed over 50634 manually tagged images from 455 TLI embryo videos and validated over a test set of 9511 images from 90 TLI embryo videos. The results are assessed by (a) detection TP and FP rates and (b) pixel-by-pixel accuracy relative to each embryo’s bonding sub-image. TP is the percentage of embryos with an Intersection Over Union (IoU) greater than 0.9 relative to some cluster of pixels with a positive score. FP is a cluster of pixels with an IoU of less than 0.5, when compared to a ground truth embryo. On the test set, the TP/FP are 0.997/0.007 with the weighting scheme applied, and 0.997/0.016 without. Using the AUC measure, the corresponding results are 0.994 versus 0.984 on the test set. The resulting per pixel 0–1 accuracy, relative to the bonding sub images that contains each embryo, is 0.976 with the weighting scheme applied, and 0.964 without. This indicates high accuracy segmentation, even without the modified loss, which is further improved (by eliminating a third of the errors) by it.

Embryo related data are highly heterogeneous and the labels are noisy. This is because the causes of infertility and implantation failure vary across cases and depend on both the embryo and the mother. Second, the Known-Implementation-Data (KID) provide partial labeling: the successful implantation cases (KIDp) indicate a viable embryo, while a negative outcome (KIDn) may indicate issues that are not necessarily associated with the embryo itself. Third, the data consist of embryo images captured at multiple time points and at different levels of development. Images taken at day 2 typically consist of 4–8 cells (known as the cleavage stage), while images taken at day 5 are typically in one of the blastocyst stages.

To tackle the first issue, we used the oocyte age as an instrumental variable since it is known to be correlated with the underlying medical challenge, and compared embryos for mothers of similar ages.

To tackle the second issue, we developed a method that relies on soft labels. This also allowed us to enable the classifier to learn from embryos for which the KID status is unknown. The soft labeling was applied to all pairs of samples $i,j\in [N]$ in a mini-batch of size *N*, containing embryos of similar oocyte age.

4 $$\begin{aligned} L= \sum _{i \ne j \in \left[ N \right] }l \left( s_{i,j},y_{i,j} \right) , \end{aligned}$$

where $s_{i,j}$ is the joint score of the pair (*i*, *j*) in a mini-batch of size N. $y_{i,j}$ is the label associated with the pair, and *l* is some loss function.

To tackle the third issue, we conditioned the prediction on the stage of development by employing multiple prediction heads in our network classifier, where each head corresponded to a temporal window of 2 h within the time interval $\Delta _{t}= \left[ t_{0},t_{e} \right]$ we inspect.

**Figure 2:**
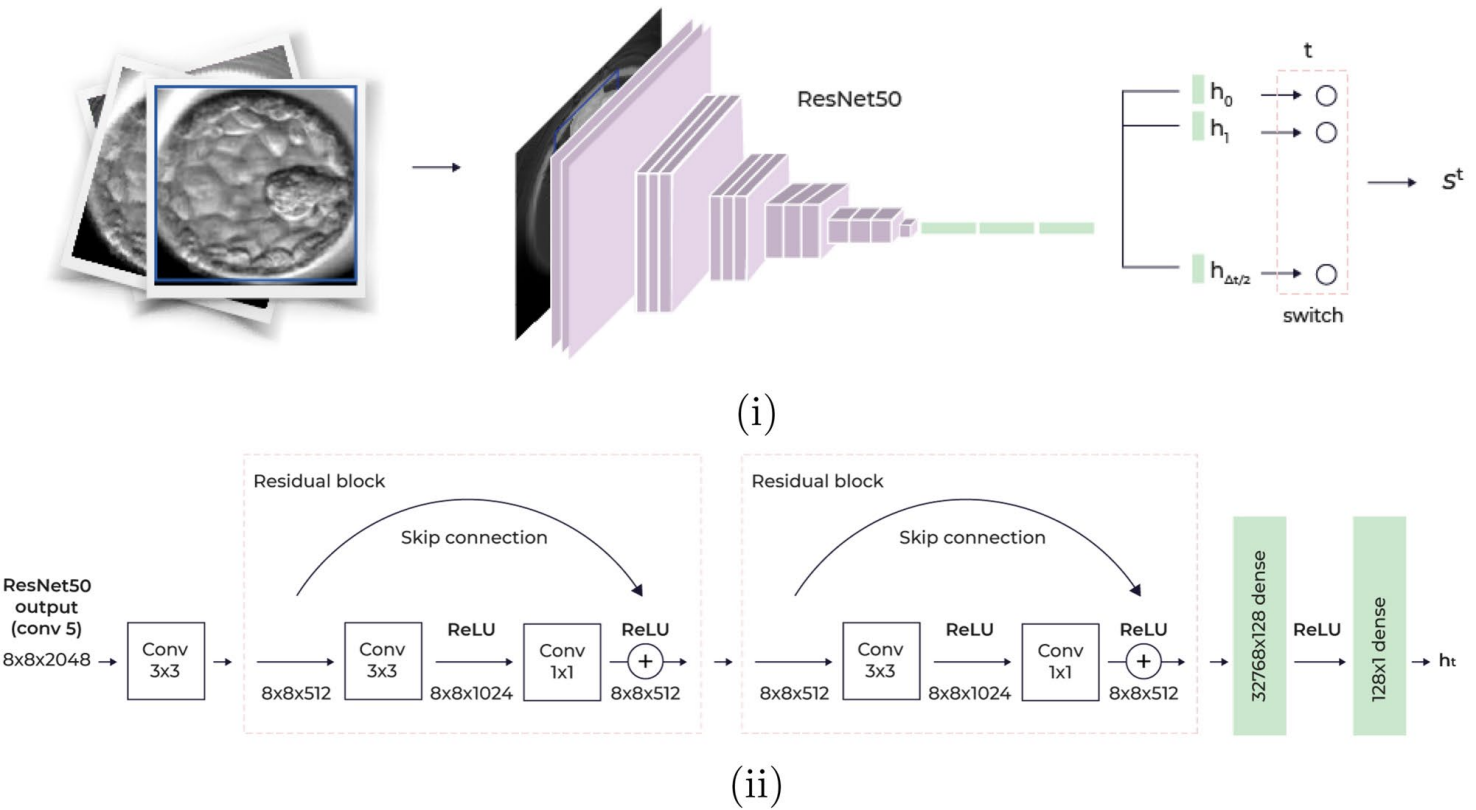
The classification model. (**i**) A $256\times 256$ cropped embryo input image, is fed into Resnet50 CNN with multiple prediction heads. Each head corresponds to a temporal window of 2 h. (**ii**) Each head accepts the last convolutional layer of ResNet50, conv5, with 2048 channels, after cumulative downsampling of the image by a factor of 16, resulting in an $8\times 8\times 2048$ image. To produce the head scalar prediction, a convolutional layer with 512 channels is followed by two residual blocks and two dense layers.

Our network models have a ResNet50^45^ backbone, after which we applied a separate classification-head of a convolution layer followed by two residual convolutions and two dense layers that are dedicated to each time window; see Fig. 2. At inference time, we only consider the head that is relevant to the frame we want to classify, thus conditioning our result on the time of capture.

To take advantage of the fact that we have multiple images for each embryo, we integrated information across different time points, making our soft labels more informative. Specifically, we considered the time interval $\Delta _{t}= \left[ t_{0},t_{e} \right]$ and integrated the classifier scores over time using an auto-regression moving average (ARMA) model, in order to obtain a fused score.

**Figure 3:**
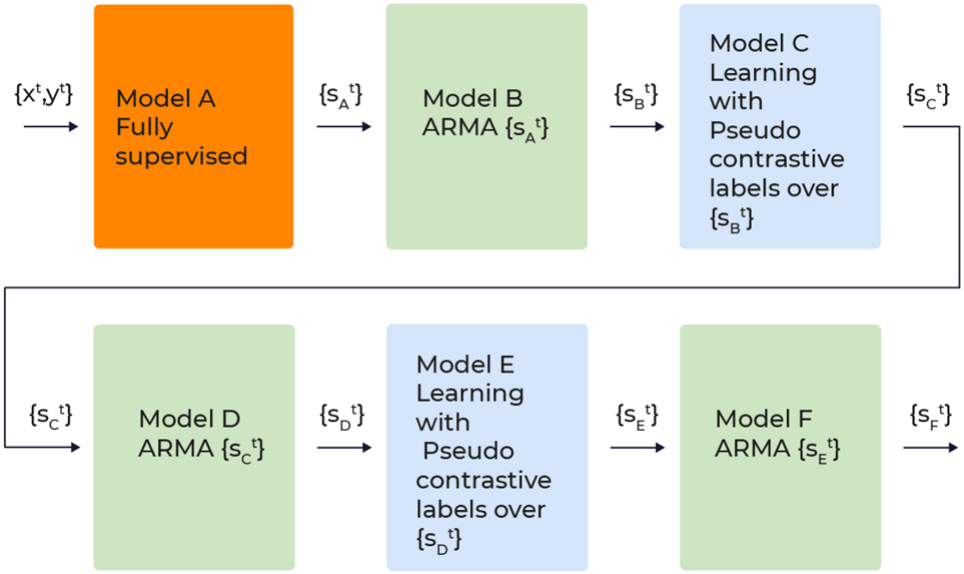
Models overview. (**A**) A CNN model, trained in a fully supervised manner over KIDp versus KIDn, a total of 7799 embryos. (**B**) A temporal ARMA filtering over the outcome of A. (**C**) A CNN model, trained using pseudo constrictive labels obtained by the outcome of B, over all training embryos, a total of 61581 embryos (**D**) An ARMA filtering over the outcome of C. (**E**) A second iteration of pseudo constrictive labels: a CNN model, trained using pseudo constrictive labels obtained by the outcome of D. (**F**) An ARMA filtering over the outcome of E.

With these building blocks, we employed six concatenated models A–F, see Fig. 3.

Model A is a binary classifier $A$ that accepts a single image $x^t$ from all time points $t \in \Delta _t$. The train data are a set of the form $\{(\{x^t_i\}_t,y_i)_i\}$ where $y_i$ is the KID label of the *i*-th embryo. The loss function is the same soft hinge loss given in Eq. (1).

5 $$\begin{aligned} L_h = \sum _{i \in [N],t\in \Delta _t} \log \left( 1+e^{\gamma \left( m-y_i A(x_i^t)\right) } \right) \,, \end{aligned}$$

where *m* and $\gamma$ are parameters that control the margin between positives and negatives and loss softness, respectively.

Model B integrates the single frame outputs $A(x_i^t)$ using a first order autoregressive-moving-average (ARMA) model^46^. Namely, for all $t \in \Delta _t$, $B(x_i^t) = \alpha A(x_i^t) + (1-\alpha ) B(x_i^{t-1})$. Embryo score $B(x_i^{t_e})$ is given by considering the last time point.

Model C is a neural network *C* that accepts an image as input. It is trained using all embryos, including those for which KID data are not available (embryos that were not transferred or for which the outcome is ambiguous).

Model B provides the pair metric $s_{i,j}$ and pair label $y_{i,j}$ from Eq. (4). The former is given for a pair of input images $x_i^{t_i},x_j^{t_j}$ as

6 $$\begin{aligned} s_{i,j} = C(x_i^{t_i})-C(x_j^{t_j}), \end{aligned}$$

7 $$\begin{aligned} y_{i,j}=\left\{ \begin{array}{ll} 1, &{}\quad B(x_i^{t_e})-B(x_j^{t_e})>\theta , y_j \ne 1\\ -1,&{}\quad B(x_j^{t_e})-B(x_i^{t_e})>\theta , y_i \ne 1 \\ 0, &{}\quad \text {otherwise} \end{array}\right. \end{aligned}$$

The CNN *C* is therefore trained based on the multi-frame scores of model B, which is applied to samples with KID positive (KIDp), KID negative (KIDn), KID unknown (KIDu), or samples with no KID tag (not-KID). These soft labels are restricted such that KIDp samples will not be on the negative side of the soft label. Note that the pairs are not limited to having the same time-stamp, which encourages early classification. The threshold $\theta$ is meant to relax errors in model B. Overly large $\theta$ may dismiss too many pairs. On the other hand , a low $\theta$ may overfit to the errors of model B.

To avoid the implantation bias that is associated with oocyte age, out of all pairs in a given mini-batch, we only considered pairs that had an oocyte age that was smaller or equal to a threshold $\theta _o$.

Model D is to model C what model B is to model A. It integrates model C’s outcome over the interval $\Delta _t$, $D(x_i^t) = \alpha C(x_i^t) + (1-\alpha ) D(x_i^{t-1})$. Model E applies the soft label procedure of Eq. (4) to model D, the same way that model C employs the outcome of model B. Finally, Model F integrates model E using the ARMA model, similar to model D with respect to C and model B with respect to A.

## Experiments

### Data

After the culturing period inside the incubator concludes, the best embryos (typically 1–2, out of 10–20) are transferred. The remaining embryos are frozen, if they appear morphologically viable, or are otherwise discarded. For those embryos that were transferred, tagging followed the Known Implantation Data (KID) designations: (i) KID-positive (KIDp), if the number of transferred embryos equals the number of gestational sacs; i.e., each transferred embryo successfully implanted, (ii) KID-negative (KIDn), if there are no gestational sacs; i.e., none of the transferred embryos are implanted, (iii) KID-unknown (KIDu), if the number of gestational sacs is greater than zero but smaller than the number of transferred embryos. In this case, the outcome of each individual embryo is uncertain.

Non-transferred embryos (frozen or discarded) have no tag and are denoted not-KID. Typically, those account for ~  85% of the embryos^39^. We denote embryos as *hard-discarded*, discarded embryos that developed at least to the blastocyst stage. Embryos that had pre-implantation genetic testing (PGT) in addition have a *euploid*/*aneuploid* tag to denote chromosomal normality/abnormality, respectively.

The data were retrospectively collected from four centers: the Ein-Kerem, and Mt. Scopus campuses of Hadassah Hebrew University Medical Center, the Soroka University Medical Center, and the NYU Langone Prelude Fertility Center. After fertilization, the embryos were incubated in EmbryoScopeTM (Vitrolife, Copenhagen, Denmark), a time-lapse incubator that captures images every 15 to 20 min. The embryo development videos were collected from July 2014 to December 2019, with oocyte ages of 23.6–43.6 years. This study was approved by the Investigation Review Board of Hadassah Hebrew University Medical Center (IRB number HMO-006-20), and by the New York University Institutional Review Board approval (IRB number S13-00389. The data were split into Train and Test, without intersection between patients, such that the test set was composed solely of embryos for which data were available for the entire culture period of 114 h, with approximately equal amounts of KIDp and KIDn for every clinic, regardless of the overall amount of data available from that clinic. This made the KIDn test set more challenging (since easier negative cases from a day 3 transfer would have been discarded and not transferred on day 5) but it made it possible to compare results between different time points on the exact same test set. The sizes of the different tag-sets for each clinic and the different splits are detailed in Tables  1 and  2, respectively.

**Table 1:** The distribution by clinic of embryo implantation and genetic data.

	KIDp	KIDn	KIDu	Discarded	Hard discarded	Not-KID	Euploid	Aneuploid
Soroka	829	3088	1006	21,862	6246	9346	0	0
Ein-Kerem	407	1714	864	6421	2058	3907	0	0
Mt. Scopus	267	1241	640	6421	2002	3058	0	0
NYU	191	809	0	5541	1885	956	401	971
Total	1694	7122	2510	38,796	12,191	17,267	401	971

**Table 2:** Train/validation (val) and Test embryo implantation/genetic data.

	KIDp	KIDn	KIDu	Discarded	Hard discarded	Not-KID	Euploid	Aneuploid
Train/val	1314	6485	2106	36,507	11,504	14,934	342	713
Test	380	637	404	2289	687	2333	59	258

The training was performed with Nvidia GeForce Titan 2080 RTX GPU, and implemented in Python using the PyTorch package.

### Setting the hyperparameters

Setting $m=0$ and $\gamma =1$ of $L_h$, the binary soft hinge loss would become the binary cross entropy loss. In our work, early on the development process we set $m=1, \gamma =1$, which added a significant margin, and was further regularized with a weight decay of 1e−5. The batch size was set to 24, thus allowing a maximum of 264 pairs in a single mini-batch. The time interval $\Delta _{t}$ was set to (30, 120), which corresponded roughly to the interval between the second split and blastocyst expansion. This was done for technical reasons since many embryos were placed in the incubator around 20–25 h, and cultured for 120 h. A head corresponded to a time window of 2 h; thus, the network consisted of 45 heads. The average oocyte age of KIDp/KIDn embryos was 31.33/35.78 with a Standard Deviation (SD) of 4.98/5.97, respectively. We employed $\theta _o = 2$, thus avoiding comparisons between oocytes that had more than one-half SD age gap, yielding an average of 32 valid pairs in a mini batch.

The parameter $\theta$ in models C and E was set by considering the two 100-bin histograms computed for the values obtained by applying model *B* to the training sets of KIDp and KIDn. Specifically, the value was the mean over the difference between the mean value of matching bins of the two histograms. A second value of $\theta$ was used in learning model E based on Model D, and was computed by considering the histograms obtained from applying model D on the two training sets.

The ARMA coefficient $\alpha$ used by integration models B,D,F was set to 0.05, to incorporate long scores memory.

Training involved all the training data, whereas the results were tested with respect to four different subsets of the test set: (i) KIDp versus KIDn, (ii) KIDp versus aneuploid, (iii) KIDp versus Discarded, and (iv) KIDp versus Hard Discarded.

**Table 3:** Classification results (AUC) for each model for the different data sets (each denoted by the negative class) at the end of day 3 and at the end of day 5.

Model	Day	KIDn	Aneuplody	Discarded	Hard-discarded
A (multi class model)	3	0.561	0.630	0.718	0.590
	5	0.653	0.827	0.916	0.849
B (integration of A)	5	0.656	0.837	0.927	0.868
C (pair model over B)	3	0.620	0.710	0.865	0.780
	5	0.669	0.869	0.965	0.924
D (integration of C)	5	0.671	0.875	0.967	0.929
E (pair model over D)	3	0.624	0.774	0.861	0.780
	5	0.678	0.897	0.967	0.935
F (integration of E)	5	**0.681**	**0.903**	**0.970**	**0.942**
Liu et al.^35^	3	0.594	0.643	0.814	0.685
KIDScore-D3^36^	3	0.582	0.641	0.832	0.707

**Figure 4:**
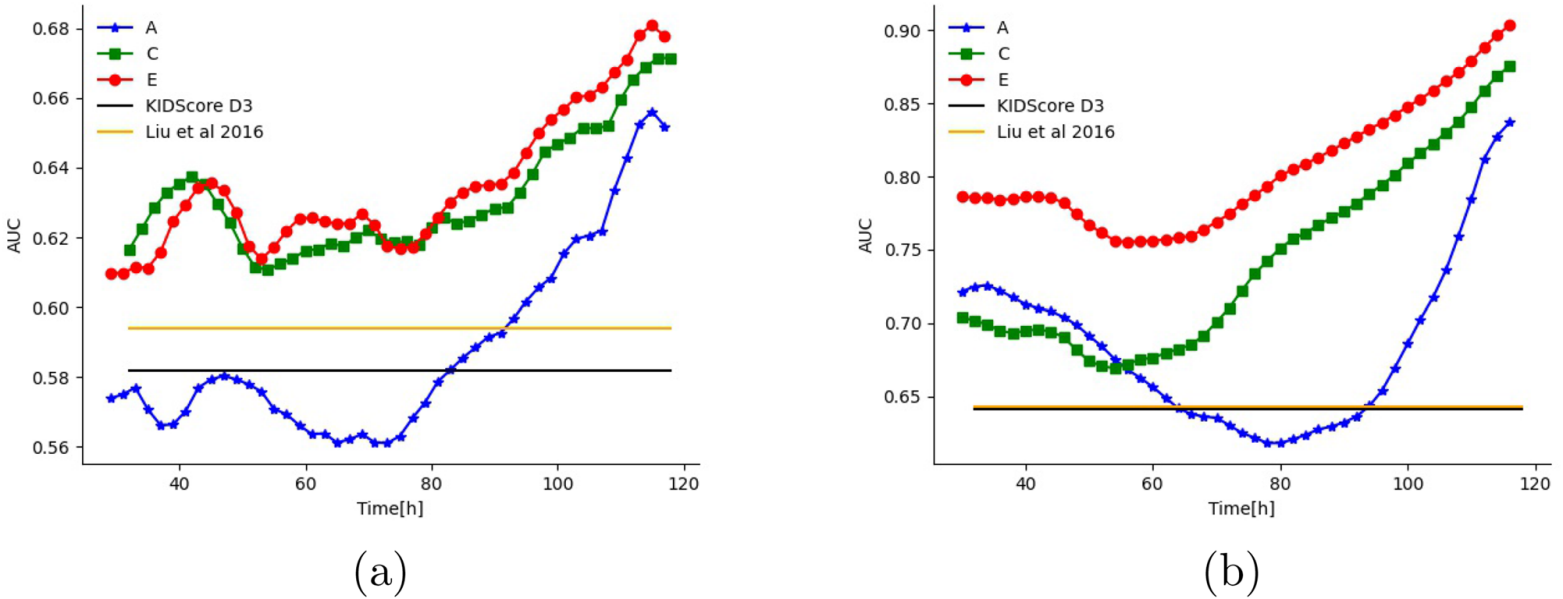
Prediction ability (AUC) as a function of time since fertilization (hours) for single-frame models. (**a**) KIDp versus KIDn classification, (**b**) KIDp versus aneuploid.

**Figure 5:**
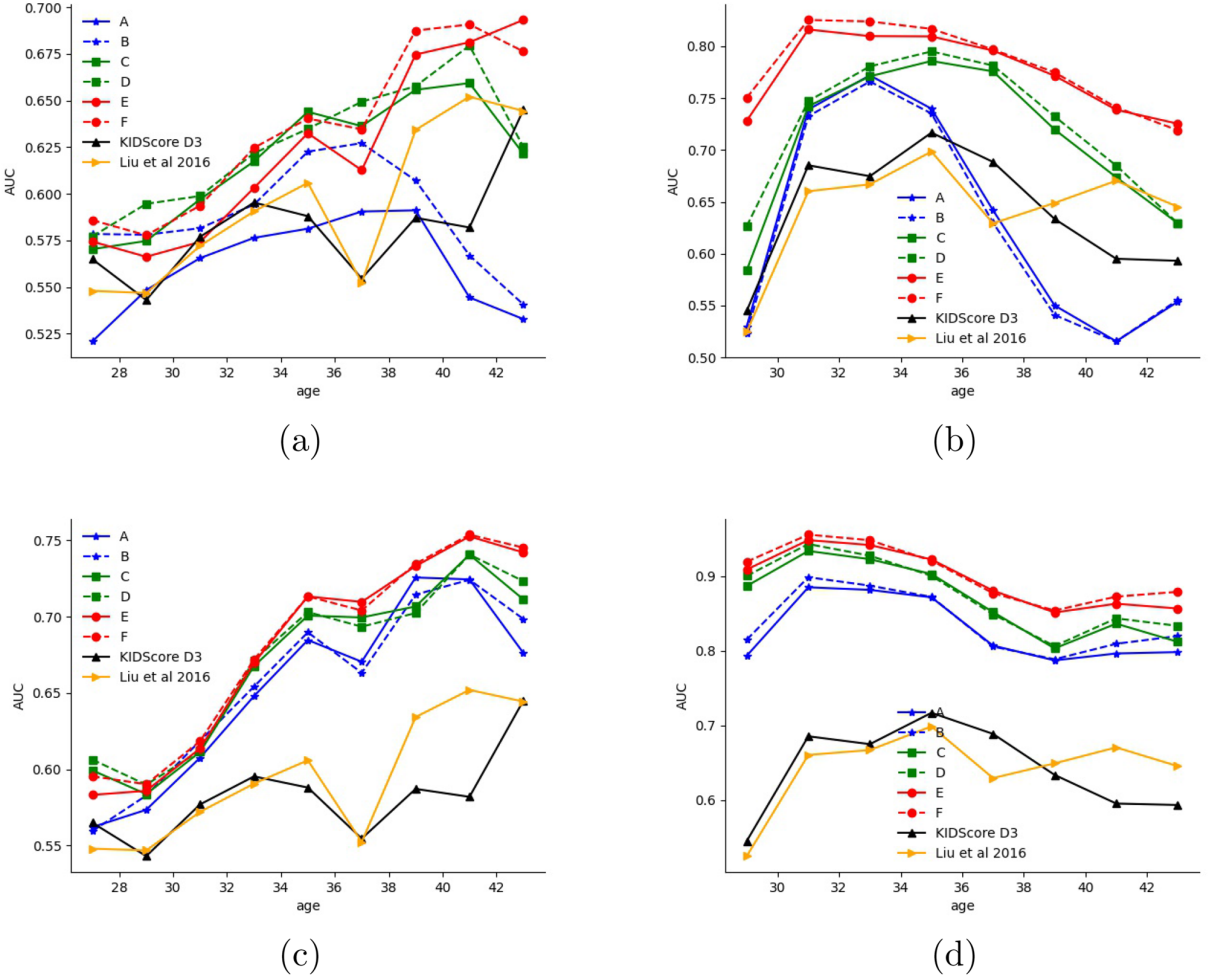
Prediction ability (AUC) as a function of oocyte age (years) for the classification of different negatives from KIDp after 3 and 5 days since fertilization (**a**) KIDp versus KIDn classification at 72 h since fertilization (**b**) KIDp versus aneuploid classification at 72 h since fertilization (**c**) KIDp versus KIDn classification at 114 h since fertilization (**d**) KIDp versus aneuploid classification at 114 h since fertilization.

We evaluated the accuracy of soft-labeling (models C–F) across all data sets, times, and ages, compared to both fully supervised learning (models A–B) and to two previously reported studies, Liu et al.^35^ and the FDA-approved KIDScore-D3^36^.

The results are reported in Table 3. Evidently, each progression of our method (from A to B to C, etc.) improved the classification accuracy, for both day 3 and day 5 and across the four classification tasks. This indicates that integration over time helps (A to B, C to D, E to F) and reinforces the utility of training the single image classifiers (C, E) using information integrated over time (B and D, respectively).

Our last single-image classifier (model E), which did not observe multiple images during test (it was trained based on sequence information), outperformed both Liu et al. and the proprietary day 3 KIDScore-D3 models on day 3, despite the fact that both latter models require 66–68 h of continuous monitoring and manual annotations.

To better understand the availability of information across different time points, we evaluated models A, C, and E at different times, see Fig. 4. The figure also shows the performance obtained by the Liu et al. and KIDScore-D3 models, after observing multiple measurements over three days. As can be seen, model E almost always improves upon model C and always improves upon model A. Both Liu et al. and KIDScore-D3 models are only competitive with our initial model (A), and with our intermediate model (C) when considering KIDp versus aneuploid (see Supplementary for the other two tasks) before 76 h since fertilization. Notably, as time progresses, the accuracy tends to increase. Around day 4, which typically corresponds to the morula stage, there is often a small drop in performance. On the contrary, starting the blastulation phase, the accuracy rate increases significantly . Both trends are well-known in the literature^47,48^.

The age of the oocyte is an indicator of the underlying fertility issues. In Fig. 5, we separate the results based on this age. As can be seen, all of our models outperform both Liu et al. and KIDScore-D3 models when predicting the KID status across all age groups, and the models that involve pair-training (C–F) outperform it in all groups for aneuploid detection as well. Interestingly, Fig. 5a,b show how the AUC gradually increases as the mother’s age increases. It is likely that these findings reflect two different mechanisms. The first is that infertility in younger patients is often due to non-embryonic causes, such as uterine cavity and receptivity of the endometrium. Thus, even if a viable embryo is transferred back to the uterus, implantation is not guaranteed. This leads to noise in the implantation labels and by extension in prediction accuracy. In contrast, older patients usually suffer from infertility due to their age, which is sometimes referred to as elderly infertility. Once a viable embryo is transferred, the likelihood of a successful pregnancy increases since there is no other cause of infertility. This results in less label noise and better accuracy. The second explanation is the diminishing number of high quality embryos with mothers’ age. Patients in their younger years normally have many high-quality embryos when considering the best one to be transferred, but with an older patient, a physician may have to pick a low-quality embryo just because there are no better options available. In turn, this makes the classification of older patients’ transfers easier. In contrast, Fig.  5c,d, which report the classification results between positively implanted (thus, also genetically viable) embryos, do not show this progressive trend because ploidy is not affected by non-embryonic factors.

See supplementary for an ablation study that compares pair loss with binary cross entropy loss.

### A comparison to human graders

The performance of multiframe model F was further compared to the ratings of eight professional embryologists, each from a different clinic, in multiple centers globally.

Each embryologist was asked to score the embryos that were transferred; i.e, KIDp versus KIDn, between 1 to 5 (higher is better). The accuracy of each score was then calculated based on the implantation tag. The results of comparing the AUC of our last model with those of the embryologists are reported in Table 4. As can be seen, the model outperforms the human embryologists across all age groups at a p-value that is 0.01 at most, often much lower.

A threshold that minimizes the cumulative False Positive (FP) and False Negative (FN) rates was chosen to compare the final model with the embryologists’ scores in a scenario suitable for clinical practice.

8 $$\begin{aligned} tH = min_{tH}(FP(tH)+FN(tH)) = \sum _{i \in [N]}{\mathbf{1}[y_i=-1]\cdot (s_i\ge tH) + \mathbf{1}[y_i=1]\cdot (s_i<tH)} \end{aligned}$$

Table 5 summarizes the best threshold for solving Eq. (8) and the corresponding False Positive, False Negative, 0–1 accuracy, Positive Predicted Value (PPV) and Negative Predicted Value (NPV).

The supplementary includes a ROC plot depicting of true positives (TP) and false positives (FP) of model F compared to the best working point of each professional embryologist at various ages. Alternatively, as suggested by DeLong et al.^49^, a nonparametric statistical approach might be taken by comparing the AUC of each embryologist with the model AUC for each age group.

**Table 4:** KIDp versus KIDn Classification results (AUC) of multiframe model F compared to professional embryologists at the end of day 5, for different ages of the mother.

Model/annotator	Age 27	Age 29	Age 31	Age 33	Age 35	Age 37	Age 39	Age 41	Age 43	Mean
Embryologist 1	0.562	0.555	0.627	0.665	0.635	0.580	0.667	0.725	0.734	0.639
Embryologist 2	0.613	0.599	0.623	0.638	0.631	0.599	0.661	0.686	0.717	0.641
Embryologist 3	0.61	0.612	0.643	0.682	0.656	0.634	0.699	0.703	0.684	0.658
Embryologist 4	0.590	0.574	0.591	0.621	0.610	0.602	0.661	0.687	0.697	0.626
Embryologist 5	0.564	0.589	0.634	0.687	0.664	0.647	0.677	0.693	0.683	0.649
Embryologist 6	0.632	0.607	0.625	0.624	0.599	0.594	0.653	0.683	0.675	0.632
Embryologist 7	0.613	0.575	0.589	0.633	0.586	0.569	0.622	0.654	0.693	0.615
Embryologist 8	0.632	0.589	0.636	0.666	0.662	0.617	0.657	0.683	0.731	0.652
Mean embryologists	**0.602**	**0.587**	**0.621**	**0.652**	**0.630**	**0.605**	**0.662**	**0.689**	**0.702**	**0.639**
SD embryologists	0.026	0.018	0.019	0.024	0.028	0.025	0.020	0.019	0.021	0.013
Model F	**0.636**	**0.638**	**0.663**	**0.703**	**0.711**	**0.687**	**0.738**	**0.760**	**0.755**	**0.699**
*p* value	0.010	0.000	0.001	0.001	0.000	0.000	0.000	0.000	0.000	0.000

The mean is computed across the nine age groups. The *p* value is obtained with the unpaired t-test.

**Table 5:** The accuracy characteristics of model F, averaged across different ages, compared with those of professional embryologists at the end of day 5, for a working point that minimizes false-positive rates.

Model/annotator	$avg\{FP+FN\}$	FP	FN	0–1 accuracy	PPV	NPV	Threshold
Embryologist 1	0.378	0.432	0.324	0.353	0.509	0.722	3.667
Embryologist 2	0.376	0.619	0.134	0.426	0.469	0.793	3.333
Embryologist 3	0.378	0.311	0.444	0.339	0.539	0.698	3.889
Embryologist 4	0.395	0.473	0.318	0.390	0.480	0.704	3.889
Embryologist 5	0.382	0.526	0.238	0.402	0.484	0.753	3.222
Embryologist 6	0.392	0.527	0.257	0.411	0.474	0.74	3.556
Embryologist 7	0.408	0.340	0.476	0.391	0.509	0.673	3.111
Embryologist 8	0.375	0.487	0.264	0.378	0.496	0.747	2.667
Mean embryologists	**0.386**	0**.464**	**0.307**	**0.386**	**0.495**	**0.729**	**3.417**
SD embryologists	0.027	0.095	0.104	0.027	0.022	0.035	0.392
Model F	**0.331**	**0.408**	**0.254**	**0.332**	**0.540**	**0.770**	**1.896**

For each score, the best threshold, False Rate, False Positive (FP), False Negative (FN), and 0–1 accuracy are reported.

## Discussion

One of the greatest challenges to successful embryo identification is the lack of labeled data. Typically, only 15% of all embryos are transferred, while others are frozen or discarded. Multiple embryos are often jointly transferred, leading to ambiguous labeling. Moreover, embryos may not implant successfully due to factors that are not embryo-related. This, in turn, means that negative labels are noisy. Here, we tackled both problems by introducing a pseudo soft labeling scheme which is able to process partial data labeling to initialize the learning process.

The resulting single-frame classifier learns a time-dependent prediction that can be applied to all embryos with no limitation of time or developmental stage. Further, it does not require any manual annotations which are time consuming, and provides continuous estimations of embryo viability as early as day 2 (Fig. 4), rather than a single score at a late time post fertilization. We show that integration of the single frame scores further improved the results. Thus, unlike other algorithms, it can be used seamlessly in real-time IVF workflows, using even low-cost conventional microscopes. Further, it was shown to outperform both the^35^ method and Vitrolife’s KIDScore-D3, in classifying KIDp versus KIDn, aneuploid, and hard-discarded embryos, at all times, starting at 30 h, in other words, 36 h before KIDScore-D3 and 38 h before Liu et al. A more detailed comparison indicated that this superiority was maintained for all ages and data sets (Fig. 5 and supplementary) for models C onward. This new single-image classifier successfully identified KIDp versus aneuploid with an AUC of 0.89, implying a potential non-invasive PGT replacement. Note that the classification of aneuploid versus KIDp is more clinically pertinent than the outcome of the PGT test itself, since not all the euploid embryos can implant. A further discussion of the results can be found in the supplementary.

## Conclusion

In the literature on fully automatic predictive IVF, only a few studies have addressed the use of multiple frames acquired over time. As far as we know, neither allows for the use of embryos with missing or ambiguous labels during training. We addressed the first problem by proposing a simple time integration method. These data are further used to train a single-frame classifier that can be used even if multiple frames are not available, learns in a semi-supervised manner that incorporates unlabeled and ambiguously labeled samples, and addresses the problem of negative implantation labels for high-potential embryos. We suggest a pseudo-labeling scheme that is applied to pairs of samples. Our results indicate that both the time integration and the pseudo labeling improve results, even when applied multiple times, in an interleaved manner. Our method exhibited considerably better performance in comparison to the existing FDA-approved system, which requires expert labeling across multiple time frames.

## Supplementary information

Supplementary Information.

## References

[1] Gardner, D. K. & Balaban, B. Assessment of human embryo development using morphological criteria in an era of time-lapse, algorithms and ‘omics’: Is looking good still important?. *Mol. Hum. Reprod.* **22**, 704–718 (2016). 10.1093/molehr/gaw057 27578774

[2] American College of Obstetricians, Gynecologists, et al. Multiple gestation: Complicated twin, triplet, and high-order multifetal pregnancy, ACOG practice bullatin no. 56. *Obstet. Gynecol.*, **104**, 869–883 (2004). 10.1097/00006250-200410000-00046 15458915

[3] Tian Y, Wang W, Yin Y, Wang W, Duan F, Zhao S (2017). Predicting pregnancy rate following multiple embryo transfers using algorithms developed through static image analysis. Reprod. Biomed. Online.

[4] Gardner DK, Lane M, Stevens J, Schlenker T, Schoolcraft WB (2000). Blastocyst score affects implantation and pregnancy outcome: Towards a single blastocyst transfer. Fertil. Steril..

[5] Chavez-Badiola A, Flores-Saiffe-Farías A, Mendizabal-Ruiz G, Drakeley AJ, Cohen J (2020). Embryo ranking intelligent classification algorithm (ERICA): Artificial intelligence clinical assistant predicting embryo ploidy and implantation. Reprod. BioMed. Online.

[6] Bori L, Paya E, Alegre L, Viloria TA, Remohi JA, Naranjo V, Meseguer M (2020). Novel and conventional embryo parameters as input data for artificial neural networks: An artificial intelligence model applied for prediction of the implantation potential. Fertil. Steril..

[7] van Loendersloot L, Repping S, Bossuyt PMM, van der Veen F, van Wely M (2014). Prediction models in in vitro fertilization; where are we? A mini review. J. Adv. Res..

[8] Raef B, Ferdousi R (2019). A review of machine learning approaches in assisted reproductive technologies. Acta Inform. Med..

[9] Hassan Md.R, Al-Insaif S, Hossain MI, Kamruzzaman J (2020). A machine learning approach for prediction of pregnancy outcome following IVF treatment. Neural Comput. Appl..

[10] Chen F, De Neubourg D, Debrock S, Peeraer K, D’Hooghe T, Spiessens C (2016). Selecting the embryo with the highest implantation potential using a data mining based prediction model. Reprod. Biol. Endocrinol..

[11] Guh R-S, Wu T-CJ, Weng S-P (2011). Integrating genetic algorithm and decision tree learning for assistance in predicting in vitro fertilization outcomes. Expert Syst. Appl..

[12] Corani G, Magli C, Giusti A, Gianaroli L, Gambardella LM (2013). A Bayesian network model for predicting pregnancy after in vitro fertilization. Comput. Biol. Med..

[13] Malinowski P, Milewski R, Ziniewicz P, Milewska AJ, Czerniecki J, Wołczyński S (2014). The use of data mining methods to predict the result of infertility treatment using the IVF ET method. Stud. Log. Gramm. Rhetor..

[14] Holte J, Berglund L, Milton K, Garello C, Gennarelli G, Revelli A, Bergh T (2007). Construction of an evidence-based integrated morphology cleavage embryo score for implantation potential of embryos scored and transferred on day 2 after oocyte retrieval. Hum. Reprod..

[15] Mirroshandel SA, Ghasemian F, Monji-Azad S (2016). Applying data mining techniques for increasing implantation rate by selecting best sperms for intra-cytoplasmic sperm injection treatment. Comput. Methods Programs Biomed..

[16] Richter KS, Harris DC, Daneshmand ST, Shapiro BS (2001). Quantitative grading of a human blastocyst: Optimal inner cell mass size and shape. Fertil. Steril..

[17] Shapiro BS, Daneshmand ST, Garner FC, Aguirre M, Thomas S (2008). Large blastocyst diameter, early blastulation, and low preovulatory serum progesterone are dominant predictors of clinical pregnancy in fresh autologous cycles. Fertil. Steril..

[18] Papanikolaou EG, Kolibianakis EM, Tournaye H, Venetis CA, Fatemi H, Tarlatzis B, Devroey P (2008). Live birth rates after transfer of equal number of blastocysts or cleavage-stage embryos in IVF. A systematic review and meta-analysis. Hum. Reprod.

[19] Khosravi P, Kazemi E, Zhan Q, Malmsten JE, Toschi M, Zisimopoulos P, Sigaras A, Lavery S, Cooper LAD, Hickman C (2019). Deep learning enables robust assessment and selection of human blastocysts after in vitro fertilization. NPJ Digit. Med..

[20] Chavez-Badiola A, Flores-Saiffe Farias A, Mendizabal-Ruiz G, Garcia-Sanchez R, Drakeley AJ, Garcia-Sandoval JP (2020). Predicting pregnancy test results after embryo transfer by image feature extraction and analysis using machine learning. Sci. Rep..

[21] Veeck LL, Zaninovic N (2003). An Atlas of Human Blastocysts.

[22] Kragh MF, Rimestad J, Berntsen J, Karstoft H (2019). Automatic grading of human blastocysts from time-lapse imaging. Comput. Biol. Med..

[23] Chen, F., Debrock, S., Peeraer, K., D’hooghe, T. & Spiessens, C. Selecting the embryo with the highest implantation potential using developmental and morphometric scoring. *Arch. Public Health* **73**, 1 (2015).

[24] Aparicio-Ruiz B, Romany L, Meseguer M (2018). Selection of preimplantation embryos using time-lapse microscopy in in vitro fertilization: State of the technology and future directions. Birth Defects Res..

[25] Meseguer M, Herrero J, Tejera A, Hilligsøe KM, Ramsing NB, Remohí J (2011). The use of morphokinetics as a predictor of embryo implantation. Hum. Reprod..

[26] Mehanna RA (2019). Cell Culture.

[27] Basile N, Caiazzo M, Meseguer M (2015). What does morphokinetics add to embryo selection and in-vitro fertilization outcomes?. Curr. Opin. Obstet. Gynecol..

[28] Hlinka D, Kal’atova B, Uhrinova I, Dolinska S, Rutarova J, Rezacova J, Lazarovska S, Dudas M (2012). Time-lapse cleavage rating predicts human embryo viability. Physiol. Res..

[29] Milewski R, Czerniecki J, Kuczyńska A, Stankiewicz B, Kuczyński W (2016). Morphokinetic parameters as a source of information concerning embryo developmental and implantation potential. Ginekol. Pol..

[30] Reignier A, Lammers J, Barriere P, Freour T (2018). Can time-lapse parameters predict embryo ploidy? A systematic review. Reprod. Biomed. Online.

[31] Swain JE (2013). Could time-lapse embryo imaging reduce the need for biopsy and PGS?. J. Assist. Reprod. Genet..

[32] Aguilar J, Motato Y, Escribá MJ, Ojeda M, Muñoz E, Meseguer M (2014). The human first cell cycle: Impact on implantation. Reprod. Biomed. Online.

[33] Scott L, Alvero R, Leondires M, Miller B (2000). The morphology of human pronuclear embryos is positively related to blastocyst development and implantation. Hum. Reprod..

[34] Li M, Zhao W, Li W, Zhao X, Shi J (2015). Prognostic value of three pro-nuclei (3pn) incidence in elective single blastocyst-stage embryo transfer. Int. J. Clin. Exp. Med..

[35] Liu Y, Chapple V, Feenan K, Roberts P, Matson P (2016). Time-lapse deselection model for human day 3 in vitro fertilization embryos: The combination of qualitative and quantitative measures of embryo growth. Fertil. Steril..

[36] Petersen BM, Boel M, Montag M, Gardner DK (2016). Development of a generally applicable morphokinetic algorithm capable of predicting the implantation potential of embryos transferred on day 3. Hum. Reprod..

[37] Gazzo E, Peña F, Valdéz F, Chung A, Bonomini C, Ascenzo M, Velit M, Escudero E (2020). The kidscoretm d5 algorithm as an additional tool to morphological assessment and PGT-A in embryo selection: A time-lapse study. JBRA Assist. Reprod..

[38] Saraeva NV, Spiridonova NV, Tugushev MT, Shurygina OV, Arabadzhyan SI, Victorovna IO (2019). Experience of using time lapse microscopy in the IVF program in patients with good ovarian reserve. Gynecol. Endocrinol..

[39] Tran D, Cooke S, Illingworth PJ, Gardner DK (2019). Deep learning as a predictive tool for fetal heart pregnancy following time-lapse incubation and blastocyst transfer. Hum. Reprod..

[40] Liao Q, Zhang Q, Feng X, Huang H, Haohao X, Tian B, Liu J, Qihui Yu, Guo N, Liu Q (2021). Development of deep learning algorithms for predicting blastocyst formation and quality by time-lapse monitoring. Commun. Biol..

[41] Ronneberger, O., Fischer, P. & Brox, T. U-net: Convolutional networks for biomedical image segmentation. In *Medical Image Computing and Computer-Assisted Intervention (MICCAI), LNCS*, Vol. 9351, 234–241 (Springer, 2015). arXiv:1505.04597 [cs.CV].

[42] Lin, T.-Y., Goyal, P., Girshick, R., He, K. & Dollár, P. Focal loss for dense object detection. In *Proceedings of the IEEE International Conference on Computer Vision* 2980–2988 (2017).

[43] Shalev-Shwartz, S., Wexler, Y. & Shashua, A. *Shareboost: Efficient Multiclass Learning with Feature Sharing*. arXiv preprint arXiv:1109.0820 (2011).

[44] Crammer K, Singer Y (2003). Ultraconservative online algorithms for multiclass problems. J. Mach. Learn. Res..

[45] He, K., Zhang, X., Ren, S. & Sun, J. *Deep Residual Learning for Image Recognition*. arXiv preprint arXiv:1512.03385 (2015).

[46] Box GEP, Jenkins GM, Reinsel GC, Ljung GM (2015). Time Series Analysis: Forecasting and Control.

[47] Alikani M, Calderon G, Tomkin G, Garrisi J, Kokot M, Cohen J (2000). Cleavage anomalies in early human embryos and survival after prolonged culture in-vitro. Hum. Reprod..

[48] Scientists A (2011). The Istanbul consensus workshop on embryo assessment: Proceedings of an expert meeting. Hum. Reprod..

[49] DeLong ER, DeLong DM, Clarke-Pearson DL (1988). Comparing the areas under two or more correlated receiver operating characteristic curves: A nonparametric approach. Biometrics.

