# Supplementary material for: Pseudo contrastive labeling for predicting IVF embryo developmental potential
Source: Sci Rep. 2022 Feb 15;12:2488. doi: 10.1038/s41598-022-06336-y (PMC8847488; doi:10.1038/s41598-022-06336-y)
Supplement: Supplementary file 1 — Supplementary Information. [file 41598_2022_6336_MOESM1_ESM.pdf]

Supplementary for Pseudo contrastive labeling for predicting IVF embryo developmental potential

I. Erlich[1,2], A. Ben-Meir[3,2], I. Har-Vardi[4,2], J. Grifo[5], F. Wang[5], C. Mccaffrey[5], D. McCulloh[5], Y. Or[6], and L. Wolf[7]

[1]The Alexander Grass Center for Bioengineering, School of computer Science and Engineering, Hebrew University of Jerusalem, Israel
[2]Fairtilty Ltd., Tel Aviv, Israel
[3]Infertility and IVF Unit, Department of Obstetrics and Gynecology, Hadassah Ein-Kerem Medical Center and Faculty of Medicine, Hebrew University of Jerusalem, Israel
[4]Fertility and IVF Unit, Department of Obstetrics and Gynecology, Soroka University Medical Center and the Faculty of Health Sciences Ben-Gurion University of the Negev, Beer-Sheva, Israel
[5]New York University Langone Prelude Fertility Center, New York, New York
[6]Fertility and IVF Unit, Obstetrics and Gynecology Division, Kaplan Medical Center
[7]The School of Computer Science, Tel Aviv University

A Additional Results

Fig. S1 and Fig. S2 are a replica of the corresponding figures in the main manuscript, with two additional negatives sets - the discarded embryos and the hard discarded embryos.

Panel (a) in fig. S1 shows how KIDp was differentiated from discarded embryos. The rationale for using this classifier was to enable a direct comparison to a human embryologist. Embryologists classify into discarded and non-discarded (i.e. KIDp+KIDn) embryos quite effectively.

The most difficult (”hard”) discarded embryos to identify were those that reached the blastulation stage, but were found to have poor morphology (such as CC graded embryos, using the Gardner method [1]). These embryos can

often only be filtered out after the morphology is clear, in the late stages of blastulation. In Panel (b), we present the identification of KIDp with the most difficult discarded embryos.

As can be seen in Fig. S1, both single-image pair models (C and E), outperformed the proprietary Day 3 KIDScore-D3 model, at all times, for hard-discarded embryos (b) and starting from 40 hours for discarded embryos (a), despite the fact that the latter required 66 hours of continuous monitoring and manual annotations. Our initial model A is on a par with KIDScore-D3 but only later (106 hours), thus demonstrating the need to deploy the other steps suggested by our method.

Fig. S2 demonstrates that all pair models (C-F) outperformed the initial models (A-B) which outperformed the KIDScore-D3 model when predicting KID status across all age groups, even at Day 3. The second integration pair model (F) outperformed the first pair integration model (D) across all ages. Further, even the latest single-image pair model (E) outperformed the first pair integration model (D) for most age groups, which emphasizes the efficiency of the second pair iteration when moving from a sequence-based decision to a single frame.

Interestingly, as can be seen from Fig. S2(a) KIDp vs. KIDn accuracy declines with age. The KIDp vs. aneuploidy, discarded and hard-discarded (panels b-d) are more stable as a function of age. This makes sense intuitively, since infertility in older patients is often the result of the lower quality of older oocytes (age related infertility) [2], whereas younger infertility cases are often associated with other background causes. Thus, typically in older patients, by selecting a good embryo, the cause of infertility is eliminated. However, for younger patients, selecting a good embryo is often insufficient for a positive outcome. This means that the KIDn label is noisier at younger ages. Aneuploid, discarded and hard-discarded scoring are purely related to the embryo, and thus are not affected by this phenomenon.

B Ablation Study

Table. S1 is an extended version of Table. 3 in the full manuscript which includes additional models, which were trained using pseudo labels but with a binary cross entropy loss instead of a pair loss.

Model G is analogous to model C in that it uses pseudo labels generated by model B, but only a multiclass loss is used. Model H integrates model G. Model I is then trained using pseudo labels generated by H. The final ablation model is analogous to model F in that it integrates the second pseudo label model, in this case model I.

As can be seen, across all data sets, Model C is better than G, D is better than H, E is better than I, and finally, F is better than J. This points to the advantages of the pairwise approach.

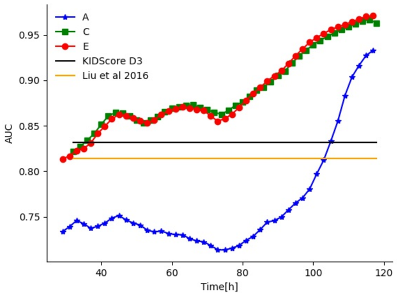

(a)

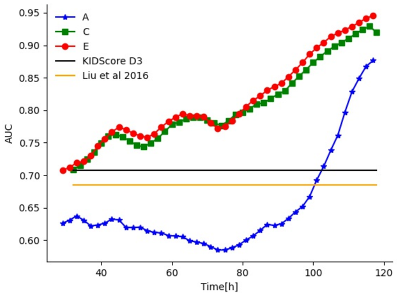

(b)

Figure S1: Prediction ability (AUC) as a function of time since fertilization (hours) for single-frame models. (a) KIDp vs. discarded, (b) KIDp vs. hard discarded.

References

[1] Gardner, D.K., Lane, M., Stevens, J., Schlenker, T., Schoolcraft, W.B.: Blastocyst score affects implantation and pregnancy outcome: towards a single blastocyst transfer. Fertility and sterility **73**(6), 1155–1158 (2000)

[2] Liu, K., Case, A., Cheung, A.P., Sierra, S., AlAsiri, S., Carranza-Mamane, B., Dwyer, C., Graham, J., Havelock, J., Hemmings, R., et al.: Advanced reproductive age and fertility. Journal of Obstetrics and Gynaecology Canada **33**(11), 1165–1175 (2011)

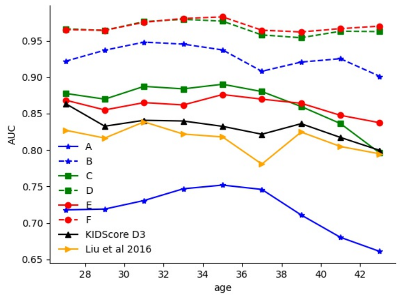

(a)

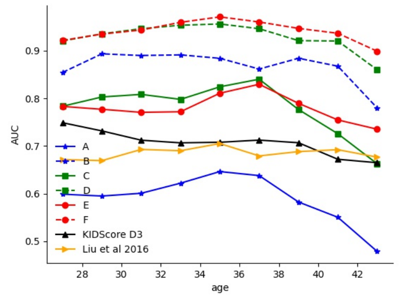

(b)

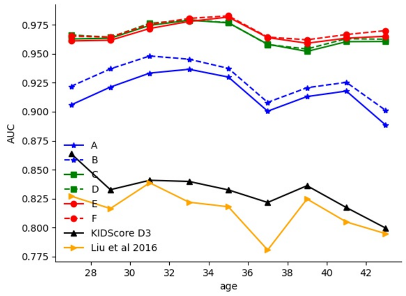

(c)

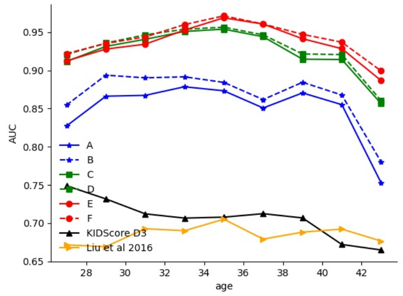

(d)

Figure S2: Prediction ability (AUC) as a function of oocyte age (years) for the classification of different discarded negatives from KIDp after 3 and 5 days since fertilization (a) KIDp vs. discarded classification at 72 hours since fertilization (b) KIDp vs. hard discarded classification at 72 hours since fertilization (c) KIDp vs. discarded classification at 114 hours since fertilization (d) KIDp vs. hard discarded classification at 114 hours since fertilization.

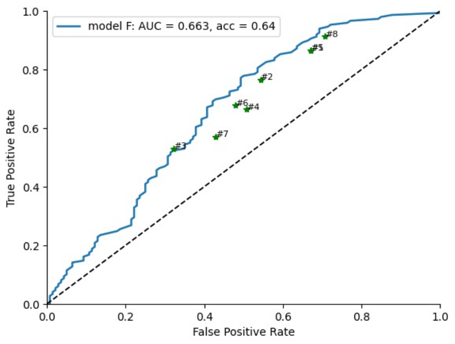

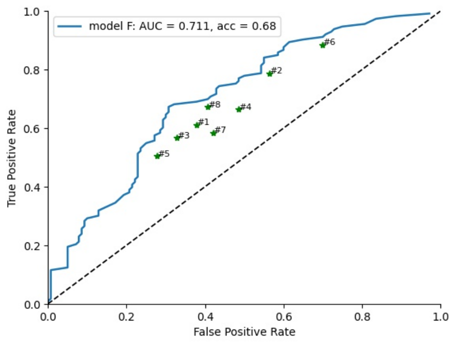

(a) (b)

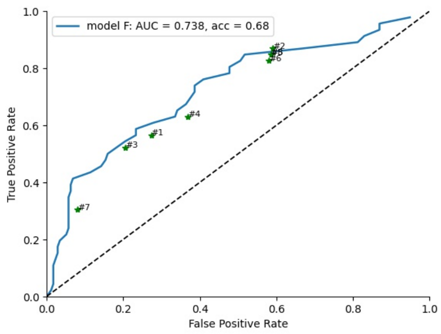

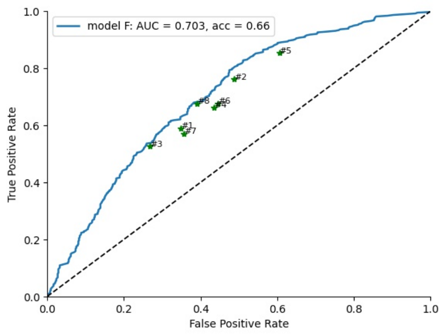

Prediction ability (ROC) of KIDp vs. KIDn classification results (AUC) compared to professional embryologists at the end of day 5, for various ages of the mother, and on average across nine age groups. Green stars indicate the best accuracy of the professional embryologists on each ROC plot. (a) age 31, (b) age 35, (c) age 39, (d) average over all ages

Table S1: Classification results (AUC) for each model, including ablations models, for the different data sets (each denoted by the negative class) at the end of Day 3 and at the end of Day 5.

Model	Day	KIDn	Aneuplody	Discarded	Hard-Discarded
A (multi class model)	3	0.561	0.180	0.718	0.590
	5	0.653	0.633	0.916	0.849
B (integration of A)	5	0.656	0.67	0.927	0.868
C (pair model over B)	3	0.620	0.604	0.865	0.780
	5	0.669	0.873	0.965	0.924
D (integration of C)	5	0.671	0.885	0.967	0.929
E (pair model over D)	3	0.624	0.724	0.861	0.780
	5	0.678	0.890	0.967	0.935
F (integration of E)	5	**0.681**	**0.904**	**0.970**	**0.942**
G (multi class model, pseudo-label over B)	3	0.565	0.360	0.744	0.683
	5	0.665	0.801	0.944	0.904
H (integration of G)	5	0.664	0.819	0.949	0.913
I (multi class model, pseudo-label over H)	3	0.605	0.662	0.836	0.740
	5	0.669	0.878	0.961	0.924
J (integration of I)	5	0.670	0.898	0.965	0.933
